# Clinical and trichoscopic features in 18 cases of Folliculotropic Mycosis Fungoides with scalp involvement

**DOI:** 10.1038/s41598-021-90168-9

**Published:** 2021-05-18

**Authors:** Giuseppe Gallo, Alessandro Pileri, Michela Starace, Aurora Alessandrini, Alba Guglielmo, Simone Ribero, Pietro Quaglino, Bianca Maria Piraccini

**Affiliations:** 1 Department of Medical Sciences, Section of Dermatology, Dermatology Clinic, University of Turin, Via Cherasco 23, Turin, Italy; 2 Division of Dermatology, Department of Experimental, Diagnostic and Specialty Medicine, S. Orsola-Malpighi Hospital, University of Bologna, Bologna, Italy

**Keywords:** Skin diseases, Skin cancer, Skin manifestations

## Abstract

Folliculotropic Mycosis Fungoides (FMF) is a rare variant of Mycosis Fungoides involving the scalp leading to alopecia. The clinical and trichoscopic features in 18 patients were analyzed and compared with the reports in the literature. Gender, age, disease stage, site of onset were taken into consideration. Clinical and trichoscopic analyses were performed on each patient. From a clinical point of view, Folliculotropic Mycosis Fungoides lesions involving the scalp presented as generalized alopecia (27.8%) or patchy-plaque alopecia (72.2%). Trichoscopic analysis revealed six most frequent features: single hair (83.3%), dotted dilated vessels (77.8%), broken-dystrophic hairs (66.7%), vellus hairs (61.1%), spermatozoa-like pattern vessels (55.6%), and yellow dots (55.6%). Additional identified trichoscopic patterns were dilation of follicular openings, scales-crusts, purpuric dots, short hair with split-end, pigtail hairs, perifollicular hyperkeratosis, milky-white globules, black dots, white dots/lines and absence of follicular dots. These trichoscopic features were further correlated to clinical presentations and stage of the disease. The rarity of the disease is a limitation. The relatively high number of patients allowed to identify several clinical and trichoscopic patterns that could be featured as specific or highly suspicious for FMF in order to consider trichoscopy as a complementary diagnostic approach and improve the differential diagnoses between FMF and other scalp disorders.

## Introduction

Mycosis Fungoides (MF) is the most common type of T-cell lymphoma and is characterized by small-medium atypical T lymphocytes infiltrating the epidermis. The clinical course of MF is typically indolent with a slow progression^1^. It can show variegate clinical presentation ranging from erythematous patches to plaques and nodules. Beyond the classical form of MF, other variants were identified as Pagetoid reticulosis, Poikilodermatous MF, granulomatous slack skin, hypopigmented or hyperpigmented MF and Folliculotropic MF (FMF)^1^. FMF is a less common clinicopathologic variant of MF with an estimated frequency of 17.8% of all MF types^2^ and 5% of all primary cutaneous lymphomas^1^. FMF variant is characterized by folliculotropic infiltration of atypical T lymphocytes, with or without follicular mucinosis or infiltration of epidermis, presented with a variable combination of follicular lesions that mainly involve the head and neck^3^. FMF can mimic several follicle-based dermatoses^4^. Although overall survival varies according to the stage of the disease, the prognosis is generally poor^5,6^. Histopathology of FMF is characterized by the presence of atypical T cell-lymphocytes invading hair follicles. Patients usually present with grouped follicular papules, acneiform lesions and indurated plaques involving the scalp and presented in various form of alopecia, which is considered as a common feature occurring in up to 65% of patients^3^. The clinical presentations of alopecia in FMF patients include scarring and non-scarring alopecia, diffuse hair loss or alopecia areata-like patterns^3,6^.

Dermoscopy is a useful tool improving diagnostic accuracy in both pigmented and non-pigmented skin lesions that permits the assessment of vascular structures and colour variants^7^. Trichoscopy is dermoscopy of hair and scalp and allows assessment of the structure of the hair shaft, the surface of the scalp, follicular openings, and superficial blood vessels^8,9^. The interest towards dermoscopy of MF and FMF has grown increasingly^7,10,11^. However, trichoscopic patterns in FMF have been described in only a few papers with a small number of patients^3,4,12^. The objective of this paper is to analyse and review the clinical and trichoscopic features in our patients with a clinico-histopathological diagnosis of FMF involving the scalp examined in two dermatology tertiary referral centres. The identification of specific dermoscopic patterns could be helpful in the differential diagnosis with other dermatoses with scalp involvement and alopecia (lupus, lichen planopilaris, alopecia areata) together with the follow-up and response evaluation in FMF patients.

## Methods

All FMF patients with scalp involvement followed at outpatient consultation of dermatology of the University of Bologna (Sant’Orsola Malpighi hospital) and the University of Torino (Città della Salute e della Scienza hospital), during 2017–2019, were reviewed in this retrospective study and compared to reports in literature. Diagnosis and staging were made according to WHO-EORTC classification for primary cutaneous lymphoma^1^. A scalp biopsy specimen was performed, if not available before, to corroborate FMF diagnosis. All patients were regularly in follow-up at the two centres. Data about gender, age, disease stage and site of onset were obtained from the patients’ charts after informed consent was obtained from every subject. Clinical and dermoscopic images were taken and stored using a computerized polarized light videodermatoscope (FotoFinder Dermoscope; TeachScreen Software GmbH, Bad Birnbach, Germany)*.* For each patient, at least 4 sites were examined: frontal-middle scalp-vertex, bilateral parieto-temporal and occipital regions. For each site, firstly, clinical images were made, then hairs were divided and trichoscopic pictures were acquired in at least two different areas with a minimum of eight pictures per patient. Regarding patients presented with patchy or plaque alopecia, both the centre and the periphery of the alopecic area was checked. Trichoscopy was carried out at first with a lower magnification (× 10) and then a higher one (× 50 or × 70) to better analyse vascular patterns and hair shaft structures. We started the examination with dry dermoscopy, and then used water as immersion fluid for an additional examination to magnify some dermoscopic features. All procedures were carried out in accordance with relevant guidelines and regulations. The study protocol was approved by the Inter-Corporate Ethics Committee of the Città della Salute e della Scienza hospital of Turin (protocol number 0072169). Statistical analyses were performed using Fisher’s test to compare clinical and trichoscopic features among groups. *P* values equal or below 0.05 were considered significant.

## Results

**Figure 1:**
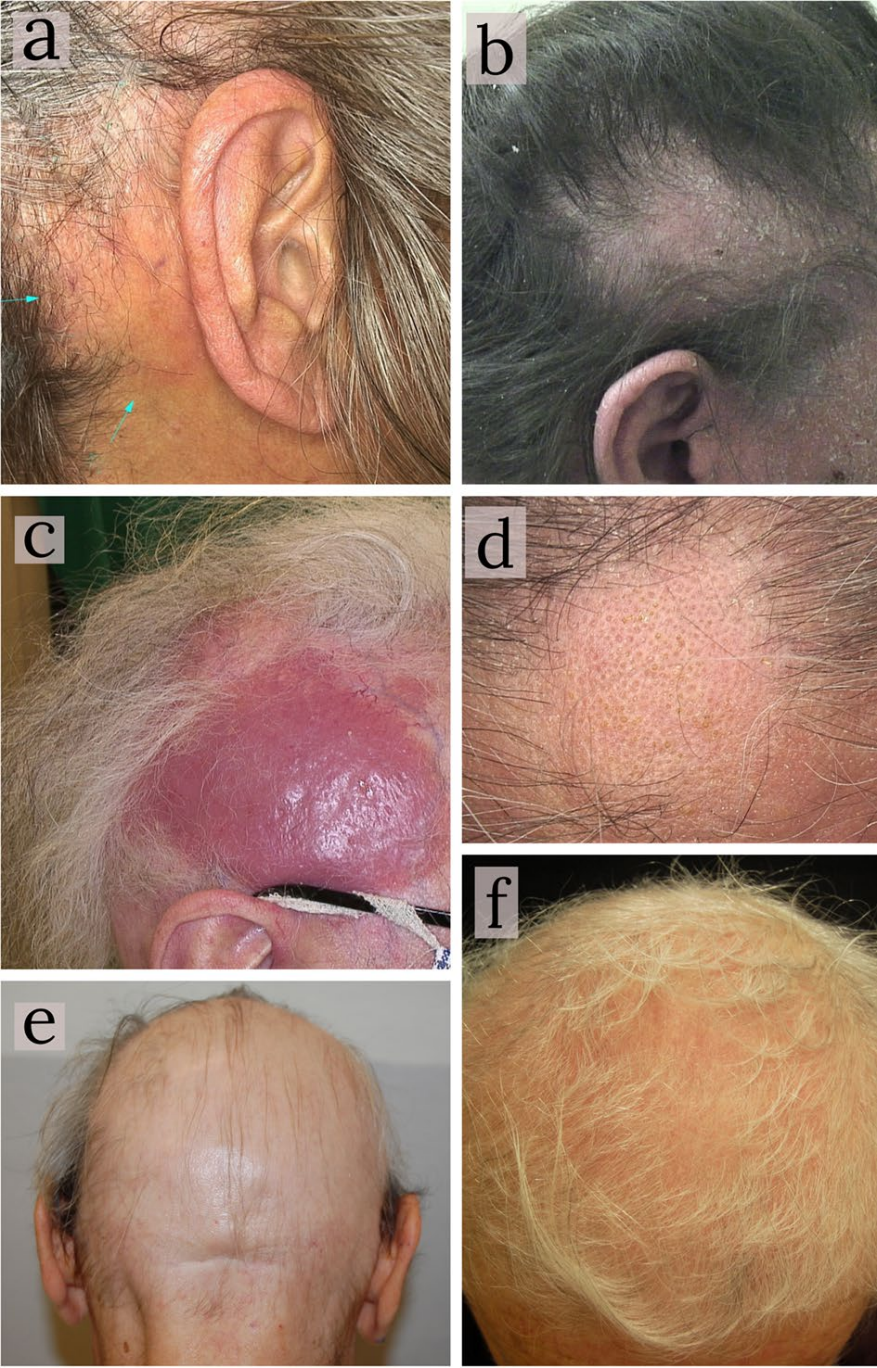
Different types of alopecia as clinical presentations of Folliculotropic Mycosis Fungoides involving the scalp. (**a**) retro-auricular erythematous patch alopecia; (**b**) scaly-erythematous patch alopecia; (**c**) erythematous plaque-nodular lesion in a patient with late Folliculotropic Mycosis Fungoides stage; (**d**) non-inflammatory frontal patchy alopecia; (**e**) generalized alopecia of the scalp in a male patient; (**f**) generalized alopecia of the scalp in a female patient.

A total of 18 patients with a previous clinico-histopathological diagnosis of FMF involving the scalp were reviewed in the study period. All patients were attending a regular follow-up at two tertiary level University hospitals, 14 patients at the Dermatology Clinic of Bologna and 4 at the Dermatology Clinic of Turin. 11 (61.1%) were women and 7 (38.9%) men. Median age of the enrolled patients was 71 (range from 51 to 97 years). Concerning staging at the time of the visit, 9 (50%) were at stage Ia, 3 at stage Ib (16.7%), 4 at stage IIb (22.2%), 2 (11.1%) at stage IIIa. In 14 (77.8%) patients, the scalp represented the first site involved by the disease (Table 1). In all 18 patients, a clinical and trichoscopic examination was performed. Alterations involved both the hairs and the surrounding skin. From a clinical point of view, FMF lesions involving the scalp were presented as generalized alopecia 27.8% (5/18) or patchy-plaque alopecia 72.2% (13/18) of patients. The latter was characterized by scaly erythematous patches (85.7%) or non-inflammatory patches (14.3%); plaque-nodular lesions occurred in 5 (27.8%) patients (Table 2) (Fig. 1).

**Table 1:** Data about gender, stage of disease and first localization of disease in our study population.

	n (%)
**Sex**	
Female	11 (61.1)
Male	7 (38.89)
**Stage of disease**	
Ia	9 (50.0)
Ib	3 (16.7)
IIb	4 (22.2)
IIIa	2 (11.1)
**First localization of disease**	
Scalp	14 (77.8)
No scalp	4 (32.2)

**Table 2:** Different alopecia patterns on a clinical point of view.

Clinical features	All patients	Early-FMF (IA–IB)	Advanced-FMF (IIB–III)
	n (%)	n (%)	n (%)
Generalized alopecia	5 (27.8)	3 (25)	2 (33.3)
Patchy-plaque alopecia	13 (72.2)	9 (75)	4 (66.7)
Non-inflammatory patchy-plaques	(14.3)	(20)	(0)
Scaly-erythematous patchy-plaques	(85.7)	(80)	(100)
Plaque-nodular lesions	5 (27.8)	5 (41.7)	3 (50)
	Tot. 18	Tot. 12	Tot. 6

**Figure 2:**
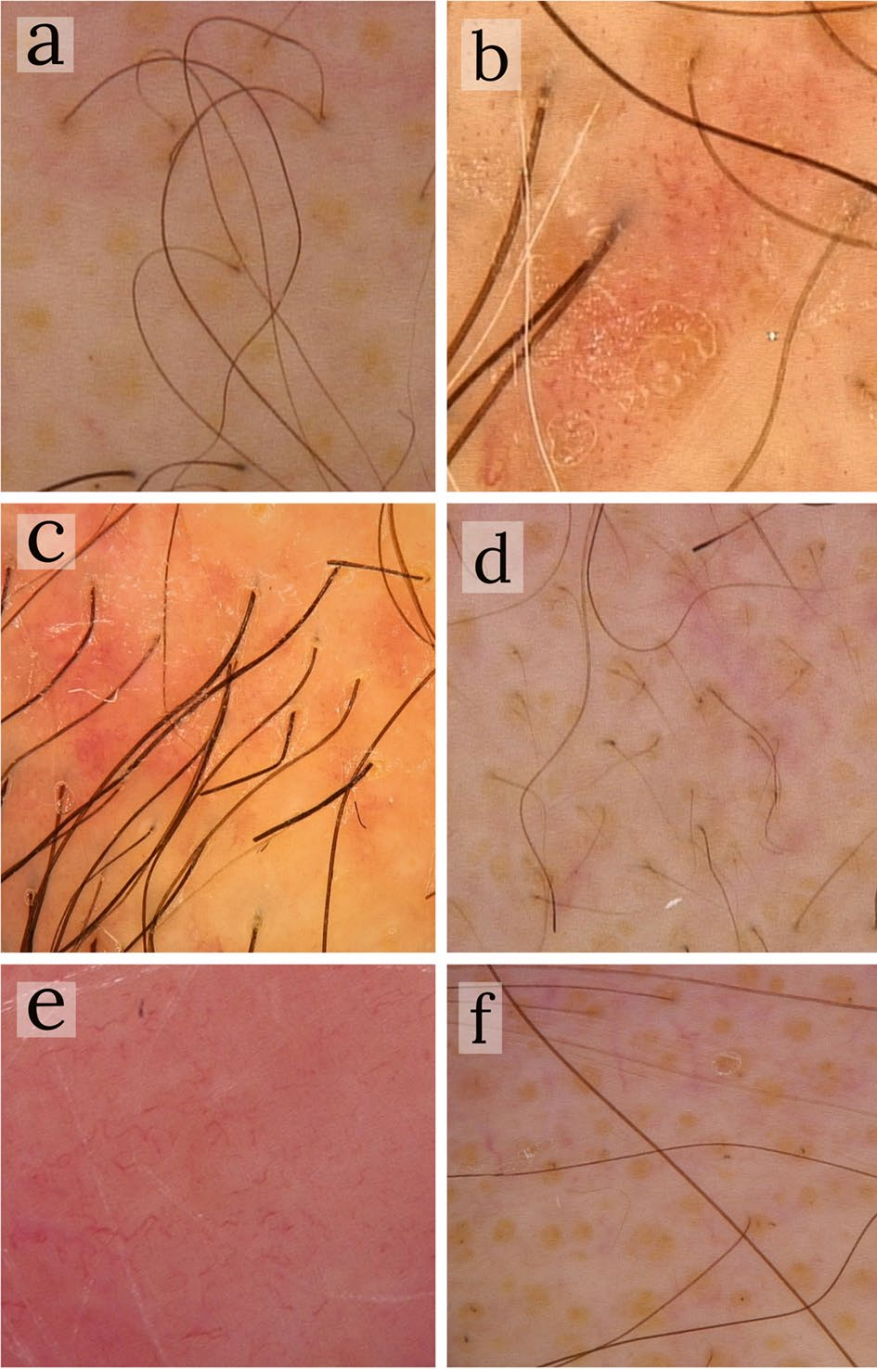
The 6 most frequent trichoscopic features in Folliculotropic Mycosis Fungoides patients. (**a**) single hairs (× 10); (**b**) dotted dilated vessels (× 50); (**c**) broken-dystrophic hairs (× 50); (**d**) vellus hairs (× 10); (**e**) spermatozoa-like pattern vessels (× 50); (**f**) yellow-dots (× 10).

**Figure 3:**
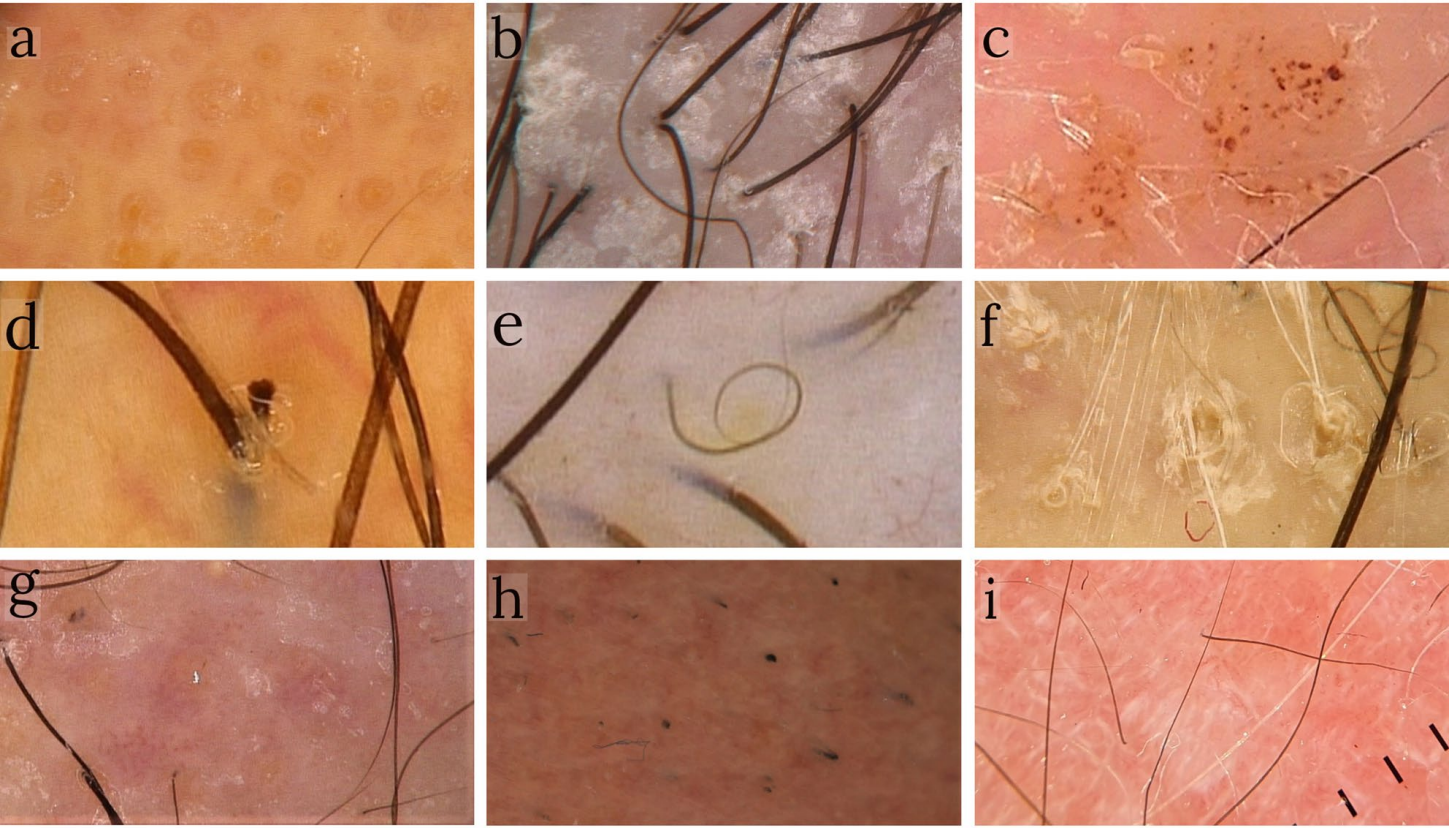
Further trichoscopic features identified in our study population: (**a**) dilation of follicular openings (× 50); (**b**) scales-crusts (× 50); (**c**) purpuric dots (× 50); (**d**) short hair with split-end (× 70); (**e**) pigtail hairs (× 50); (**f**) perifollicular hyperkeratosis (× 70); (**g**) milky-white globules (× 50); (**h**) black dots (× 50); (**i**) white dots/line and absence of follicular dots (× 10).

Trichoscopic examination revealed 6 features referable to non-scarring alopecia present in more than half the patients: decreased number of pilosebaceous units (single hair) in 83.3% (15/18), dotted dilated vessels 77.8% (14/18), broken-dystrophic hairs 66.7% (12/18), vellus hairs in 61.1% (11/18), spermatozoa-like pattern vessels 55.6% (10/18), and yellow-dots 55.6% (10/18) of patients (Fig. 2). Other features, referable to both non-scarring (dilation of follicular openings, scales-crusts, purpuric dots, short hair with split-end, pigtail hairs, perifollicular hyperkeratosis, milky-white globules, and black dots) and scarring alopecia (white dots/line and absence of follicular dots replaced by fibrosis) (Fig. 3) were present in a minority of patients (from 16.6% (3/18) to 44.4% (8/18) (Table 3).

**Table 3:** Trichoscopic features of study population and correlations to clinical presentations and stage of disease.

Trichoscopic features	All patients	Generalised alopecia	Patchy-plaque alopecia	Patchy alopecia	Plaque alopecia	Early-FMF (IA–IB)	Advanced-FMF (IIB–III)
	n (%)	n (%)	n (%)	n (%)	n (%)	n (%)	n (%)
Single hairs	15 (83.3)	4 (80)	11 (84.6)	7 (87.5)	4 (80)	11 (91.7)	5 (83.3)
Dotted dilated vessels	14 (77.8)	5 (100)	9 (69.2)	5 (62.5)	4 (80)	10 (83.3)	4 (66.7)
Dystrophic hairs	12 (66.7)	3 (60)	9 (69.2)	6 (75)	3 (60)	6 (50.0)	6 (100)
Vellus hairs	11 (61.1)	4 (80)	7 (53.8)	4 (50)	3 (60)	6 (50.0)	4 (66.7)
Spermatozoa-like vessels	10 (55.6)	3 (60)	7 (53.8)	4 (50)	3 (60)	8 (66.7)	2 (33.3)
Yellow dots	10 (55.6)	3 (60)	7 (53.8)	5 (62.5)	2 (40)	8 (66.7)	2 (33.3)
Dilation of follicular openings	8 (44.4)	2 (40)	6 (46.2)	4 (50)	2 (40)	6 (50.0)	2 (33.3)
Scales-crusts	8 (44.4)	2 (40)	6 (46.2)	5 (62.5)	1 (20)	4 (33.3)	4 (66.7)
White dots/lines	6 (33.3)	2 (40)	4 (30.8)	3 (37.5)	1 (20)	2 (16.7)	4 (66.7)
Short hair with split end	5 (27.8)	0 (0)	5 (38.5)	3 (37.5)	2 (40)	2 (16.7)	3 (50)
Pigtail hair	5 (27.8)	1 (20)	4 (30.8)	4 (50)	0 (0)	5 (41.7)	0 (0)
Purpuric dots	5 (27.8)	1 (20)	4 (30.8)	2 (25)	2 (40)	3 (25.0)	2 (33.3)
Absence of follicular dots	4 (22.2)	2 (40)	2 (15.4)	2 (25)	0 (0)	1 (8.3)	3 (50)
Perifollicular hyperkeratosis	4 (22.2)	1 (20)	3 (23.1)	3 (37.5)	0 (0)	3 (25.0)	1 (16.7)
Black dots	3 (16.7)	1 (20)	2 (15.4)	1 (12.5)	1 (20)	1 (8.3)	2 (33.3)
Milky-white globules	3 (16.7)	1 (20)	2 (15.4)	1 (12.5)	1 (20)	1 (8.3)	2 (33.3)
	(Tot = 18)	(Tot = 5)	(Tot = 13)	(Tot = 8)	(Tot = 5)	(Tot = 12)	(Tot = 6)

Trichoscopic features were then analysed according to the two clinical subgroups (generalized alopecia and patchy-plaque alopecia). Both groups presented the same 6 features represented in more than half the patients (dotted dilated vessels, decreased number of pilosebaceous units/single hair, vellus hair, spermatozoa-like pattern vessels, yellow-dots, broken\dystrophic hairs). There were no differences between the two groups regarding the less frequent features except for short hair with split-end and absence of follicular dots: short hair split-end was not found in patients with generalised alopecia but was observed in 38.5% (5/13) of patchy alopecia cases (*p* = 0.25); on the other hand, follicular dots were absent in 40% (2/5) of generalised alopecia and in only 15.4% (2/13) of patchy alopecia patients (*p* = 0.5) even if both these differences were not statistically significant (*Fisher’s test*) (Table 3).

Based on the clinico-histological analysis, patchy-plaque alopecia cases were furthermore distinguished in patchy and plaque alopecia pattern, and trichoscopic features were then analysed according to the two groups. Regarding the distribution of pigtails (present in 50% (4/8) of patchy alopecia and always absent (0/5) in plaque alopecia), perifollicular hyperkeratosis (37.5% (3/8) vs. 0, respectively) and absence of follicular dots (25% (2/8) vs. 0) differences were found even if not statistically significant (*p* = 0.1, *p* = 0.23 and *p* = 0.49, respectively). Moreover, patchy alopecia patients showed scales and crusts more frequently (62% (5/8) vs. 20% (1/5) respectively) (*p* = 0.27), yellow dots (62.5% (5/8) vs. 40% (2/5)) (*p* = 0.59), perifollicular hyperkeratosis (37.5% (3/8) vs. 0) (*p* = 0.23), and absence of follicular dots (25% (2/8) vs. 0) (*p* = 0.49). No other differences could be identified between the two groups (Table 3).

Finally, all patients, staged according to WHO-EORTC, were grouped as early-FMF (stage IA-IB) and advanced-FMF (IIB-IIIA). From a clinical point of view, both groups shared a common patchy-plaque pattern (Table 3). Regarding trichoscopy, both groups shared single hairs and dotted dilated vessels as more frequent features. With respect to the advanced patients, early-FMF ones were characterized by pigtail hairs (41.7% (5/12) vs. 0) (*p* = 0.11), yellow dots and spermatozoa-like pattern vessels (66.7% (8/12) vs. 33.3% (2/6)) (*p* = 0.32), even if no statistically significant differences were found. On the other hand, advanced-phase FMF showed higher prevalence of broken hairs (*p* = 0.05), white dots lines (*p* = 0.1) and absence of follicular dots (*p* = 0.08) (Table 3).

## Discussion

We report here the first trichoscopic analysis in a case series of FMF patients with scalp involvement. Dermoscopy, thanks to the visualization of features not visible to the naked eye, which can be regarded as an intermediate step between clinical examinations and dermatopathology^13^: thus, dermoscopy in general dermatology has become a very increased technique for non-invasive diagnoses.

Concerning lymphoid cutaneous lesions, most literature to date has focused upon MF, identifying peculiar cutaneous dermoscopic patterns: dotted or fine short linear vessels, spermatozoa-like structures, orange-yellowish patchy areas, comedo-like openings, white structureless areas^7,10,11,14^. A recent paper by Rakowska et al.^15^ analysed the trichoscopic patterns in a large group of erythrodermic cutaneous T-cell lymphoma reporting pili torti (81%), broken hairs (75%), eight-shaped hairs (19%), black dots (25%), yellow dots (87%), linear perifollicular (31%), glomerular (50%), dotted (25%) and arborizing vessels (31%), white thick interfollicular bands (56%), patchy hyperpigmentation (38%), white interfollicular scaling (88%), follicular spicules like scaling (13%) as the main represented features.

However, only a few papers described trichoscopic patterns in FMF. Slawinska et al.^3^ described the presence of decreased numbers of pilosebaceous units, milky-white globules, yellow dots with or without black dots/broken hairs, short hair with split-end, short hair with triangular-shape end, short, broken hair and pigtail hairs appearance hair and some areas with white dots and lines corresponding to hair follicles replaced by fibrosis. In the Atlas of Trichoscopy by Rudnicka et al.^16^, similar features were reported, in particular milky-red globules, orange-yellow patchy areas, the vascular granular well-margined pattern, milky-red areas and comedonal lesions within alopecic patches. Souissi et al.^17^ described a peculiar trichoscopic pattern in a patient with early scalp manifestations of FMF with coats of keratinaceous debris around follicle openings and multiple keratotic cone-shaped spicules surrounding follicular openings.

Our case-series evaluated the most common trichoscopic patterns in FMF scalp involvement, most of them in common with those observed in the other studies, although adding additional observations about the trichoscopic spectrum in FMF. Based on clinical features, our results indicated that most FMF patients with scalp involvement is presented as patchy-plaque alopecia (72.2%), mostly with scales-erythematous patches (85.7%) instead of generalized alopecia (27.8%). It also exhibited a combination of characteristic trichoscopic features consisting of a decreased number of pilosebaceous units (single hair), dotted dilated vessels, dystrophic hairs, vellus hairs, spermatozoa-like pattern vessels and yellow-dots in more than half (range from 83.3 to 55.6%) of our patients. Less frequent trichoscopic features were dilation of follicular openings, scales-crusts, white dots/lines, purpuric dots, short hair with split-end, pigtail hair, absence of follicular dots, perifollicular hyperkeratosis, milky-white globules, and black dots (range from 44.4 to 16.7%).

In this study, we correlated trichoscopic findings with the clinical features comparing generalized versus patchy-plaque alopecia, patchy versus plaque and early versus advanced FMF stages. Even if the present study evaluated trichoscopic features in the largest FMF series, the relatively low number of patients limited the power of statistical analysis. Despite these limitations, we considered that relevant features were highlighted by our study. Comparing generalized alopecia patients with patchy-plaque alopecia patients, dotted dilated vessels, decreased number of pilosebaceous units, dystrophic hairs, spermatozoa-like pattern vessels, yellow-dots and vellus hair came out as the most frequent features present in both groups. The main differences were noticed for short hair with split-end, totally absent in generalized alopecia patients, and absence of follicular dots: according to this data, generalized alopecia pattern could be considered a more advanced stage of the patient’s disease history with features more frequently related to scarring alopecia instead of active inflammatory disease.

In patients with patchy-plaque alopecia, trichoscopic patterns were further distinguished in patches alopecia and plaque alopecia. Pigtail hair, absent in the plaque group but present in half of the patients with patchy alopecia, resulted as the most relevant difference. Scales-crusts and perifollicular hyperkeratosis emerged more frequently in patches alopecia, indicating that the scaly component is more represented in patches lesions than plaque ones.

Finally, the trichoscopic features were analysed according to two disease stages groups: early-FMF and advanced-FMF. From our data, pigtail hairs, yellow-dots and spermatozoa-like pattern vessels resulted more frequently in early than advanced-FMF stages, indicating that the signs of follicular invasion and inflammation were prevalent in the early stages of the disease. In fact, the presence of pigtail hairs was confirmed to characterize early phases of disease, as it was also found more frequently in patchy with respect to plaque alopecia. On the other hand, scarring alopecia features, like white dots and absence of follicular dots generally replaced by fibrosis, were more frequent in advanced-FMF, indicating a more aggressive behaviour of the disease with more scarring.

The relevance of trichoscopy in FMF can be considered as twofold. From one site, it could constitute an ancillary methodology for diagnostic purposes. In fact, the detection of the 6 features, which we found to be represented in more than half the patients (dotted dilated vessels, decreased number of pilo-sebaceous units/single hair, vellus hair, spermatozoa-like pattern vessels, yellow-dots, broken\dystrophic hairs), in accordance with literature data and previous authors’ findings^3,10,16,18^, could be considered suggestive of FMF scalp involvement. In particular, early stages of the disease could be difficult to differentiate from other inflammatory skin disorders^7^ but the detection of the observed patterns might represent a very early sign that would allow clinicians to consider a biopsy and choose a proper biopsy site through dermoscopy-guided biopsy, especially in patients with follicular lesions behaving unexpectedly or not responding to treatment. However, trichoscopic patterns in FMF showed numerous overlapping features, demonstrating variability in clinical and dermoscopic presentations. Lesions could mimic a variety of follicle-based dermatoses including infections or non-infectious diseases like folliculitis, acneiform dermatoses, keratosis pilaris, lichen spinulosus or other forms of scarring and non-scarring alopecia^4^. Nevertheless, some trichoscopic findings in FMF like dilation of follicular openings^12^ and spermatozoa-like vessels^10^ were considered specific because they were so unusual in other conditions and helped differentiating disorders.

Thus, trichoscopy can be useful in assisting when differentiating FMF scalp lesions from other scalp alopecia, confirming a possible diagnostic role of trichoscopy in FMF.

From the other site, trichoscopy can constitute an adjunct modality to follow-up better the clinical course of the disease and the evolution of the lesions, which shows a prevalence of inflammatory and non-scarring alopecia features in the early phases (pigtail hairs, yellow-dots and spermatozoa-like pattern vessels, scales-crusts and peri-follicular hyperkeratosis) whilst mainly developing the characteristics referable to cicatricial alopecia in the advanced stages (white dots and absence of follicular dots). In this scenario, a further step could be to analyse the trichoscopic findings vis-a-vis with the histopatological features and degree of infiltration. Indeed, recent studies from Hodak et al.^19^ and the Dutch group^5^ showed that FMF can be presented with 2 distinct patterns, the early-stage with follicle-based patch/flat plaques, keratosis pilaris-like lesions, acneiform lesions (patch FMF) and a good prognosis similar to early-stage classic MF and the advanced stage with follicle-based infiltrated plaques and/or tumours (plaque FMF). The distinction between flat and infiltrated plaques could only be carried out on a histopathological background based on the extent and depth of the atypical lymphoid infiltrate. The identification of specific trichoscopic features able to differentiate between thin and thick plaques could be an ideal tool to discriminate the two subtypes without the need of a histological sampling. This could not be done in the present study due to the low number of patients with plaque lesions (only 5) and the lack of histopatological samples available performed in clinically selected areas.

## Conclusions

Trichoscopy, in addition to clinical observation, could confirm the diagnostic suspect of Folliculotropic Mycosis Fungoides and allow the clinician to improve the differential diagnoses with other scalp disorders and to identify more accurately suspicious features to proceed earlier with biopsy. According to our case study, in about 80% of patients the scalp can be the first site involved by the disease so clinicians with a suspicion of a patient with scalp FMF should consider extending the clinical exam to all body skin in order to identify other disease lesions and localizations.

Further studies will be needed to clarify the sensitivity and specificity values of the dermoscopic features analysed.
